# Occupational burnout in information technology workers

**DOI:** 10.3389/fpubh.2026.1791615

**Published:** 2026-05-28

**Authors:** Edlin Garcia Colato, Sagar Samtani, Jonathan T. Macy

**Affiliations:** 1 Department of Health & Wellness Design, School of Public Health-Bloomington, Indiana University, Bloomington, IN, United States; 2 Department of Operations & Decision Technologies, Kelley School of Business, Indiana University, Bloomington, IN, United States; 3 Department of Applied Health Science, School of Public Health-Bloomington, Indiana University, Bloomington, IN, United States

**Keywords:** burnout, job demand resources model, mental health, mental health literacy, occupational health

## Abstract

**Introduction:**

Burnout in the information technology sector is a growing concern, especially as information technology permeates all industries, increasingly serving as the backbone of modern organizations. Leadership is also responsible for supporting information technology teams and individuals. However, information technology professionals possess unique job responsibilities (e.g., on-call, technical complexities). This study aims to (1) assess burnout differences between information technology professionals in leadership and associate-level roles and (2) test for differences in information technology leadership and associates' assessment of their mental health literacy.

**Methods:**

This cross-sectional study was conducted between February and May 2023. A total of 462 US-based information technology professionals responded to the IT MindState online survey, of which 292 who completed the Burnout Assessment Tool were included in the analyses. Descriptive statistics (means, standard deviations, frequencies) were calculated. Mann-Whitney U tests were used to compare burnout and mental health literacy between leadership and associates-level professionals.

**Results:**

There was no statistically significant difference in burnout risk between associate-level and leadership/management. However, leadership reported significantly higher mental health literacy (*z* = −4.097, *p* < 0.001) compared to associates-level respondents.

**Discussion:**

Associates have similar burnout scores to their leadership counterparts, however their knowledge of mental health symptoms and ability to recognize them is significantly lower. These findings suggest that mental health literacy, an important resource for symptom recognition, is insufficient to buffer the demands of the IT sector. Therefore, interventions focused on the organization rather than the individual could help to reduce systemic stressors.

## 1 Introduction

Nearly two-thirds of US adults report work-related burnout (1). Occupational or workplace burnout “is an exhaustion due to the prolonged exposure to work-related problems” (2). While individuals in every sector are susceptible to workplace burnout, there are some job conditions that have been conventionally known to lead to stress. Common stressors include management style, career concerns, the design of tasks, and environmental conditions such as noise and pollution (3). Unaddressed workplace burnout results in presenteeism, absenteeism, turnover, and resignation. Costs from the employer's perspective for unaddressed burnout per employee can range from nearly $4,000 (non-manager hourly) to nearly $21,000 (executive) each year (4).

Information Technology (IT) in particular is a sector that has grown over the decades, and underpins many modern organizations in their day-to-day operations. Roles such as cloud solutions architects, cybersecurity analysts, systems administrators, and others have become a mainstay and popular occupations. Over four decades ago, stress levels among IT professionals were reportedly lower than those of other professions (5). However, today the IT sector not only encompasses nearly every industry, but the sources of vulnerabilities and threats they work to counter have also increased (6). News articles and industry reports are sounding the alarm about the high turnover and burnout occurrences among IT professionals (7–9).

The academic literature examining burnout and other mental health concerns for IT professionals has evolved over the decades. Studies from the 1990s include a longitudinal study with 10 male software engineers assessing stress and depression in Japan (10, 11). The study showed events at work were correlated with changes in depressive symptoms and stress. In reviewing the biochemical markers, the researchers found adrenaline levels increased during “new” events or as a major deadline approached, while cortisol (a marker of stress) levels increased after the conclusion of major work events. By 2006, there was already an updated view which led to mapping efforts to further understand burnout among IT professionals, tuning into the specific IT occupation rather than the general workforce (12). Recent literature explores the risk factors associated with different health outcomes by comparing the UK's IT workforce to all other professions in the UK, finding sedentary work was higher among the IT workers (13). The findings were drawn from the UK Biobank Cohort data (ages of 37 and 73), where the IT professionals in the sample had a reported median age of 50. Within their IT subgroup, IT technicians experienced more anxiety, depression, and loneliness compared to the IT managers. However, it is unknown whether burnout differed between the two groups because it was not included in the UK Biobank Cohort study.

Risk of burnout is a major concern among IT professionals, making up a significant portion of shared information online (14). Especially considering that effort-reward imbalance contributes to burnout among IT professionals (15). Best practices in organizations include having supportive leadership who participate in trainings such as mental health awareness (16), to be able to protect, promote, and provide essential resources to their employees (17). Knowledge and beliefs about risk factors and causes are a major component of mental health literacy (MHL). MHL has four dimensions that capture a person's knowledge and beliefs about mental health and knowing when and where to seek help when needed to help prevent mental health disorders and maintain one's mental health (18). MHL emerges through targeted evidence-based trainings, such as Mental Health First Aid (19), serving as a prevention tool helping individuals to identify symptoms early.

The research focused on IT MHL is limited, with a recent article identifying that the majority of online content shared amongst IT professionals is related to “Knowledge and beliefs about risk factors and causes, self-treatments/interventions, and professional help available” and major spikes in information sharing are focused on burnout (14). Since it was a study focused on social media content, the difference between IT leadership and associate level MHL was not available.

The Job Demands-Resources (JD-R) model suggests there are two major categories that impact workers: work demands and work resources (20). Work-related demands when requiring excess effort are related to burnout, specifically exhaustion, while insufficient job resources are associated with disengagement at work. Typically, there is a distinct expectation in job demands and available resources that is evident between employees in non-managerial and leadership/management roles (21). The JD-R Model has been previously used in other IT burnout research exploring differences in gender (22), and how job demands and resources influence burnout (23). Burnout, captured via turnover rates, was higher among women compared to male IT professionals (22). In assessing burnout among IT professionals, all dimensions of burnout were impacted by supervisor support, a job resource (23), further substantiating the important role leadership plays in workplace wellbeing. Job tasks, role conflict, and role ambiguity are major contributors of burnout among IT professionals (24). Furthermore, considering MHL has been shown to have a positive relationship with workplace wellbeing in other groups (25, 26), it can also be viewed as a resource. Under the JD-R framework, differences between leadership and associate roles often result in disparities of demands and resources. Since MHL functions as a resource, this too could vary across these two organizational hierarchical levels.

The aims of this study were to 1) explore burnout among IT professionals and identify any differences between IT professionals in leadership/management roles and those in associate-level roles and 2) compare IT leadership/management and IT associates' assessment of their MHL and help-seeking intentions. The hypotheses were that similar to previous studies on burnout among IT professionals, a) IT associates would report higher levels of burnout when compared to leadership/management, and b) IT leadership/management would report higher knowledge about mental health information compared to IT associates. This study is guided by the Job Demands-Resources (JD-R) Model (21) and MHL (18) as a conceptual lens rather than a framework being tested as the variables in the study do not include specific job demands.

## 2 Materials and methods

### 2.1 Study design and data collection

#### 2.1.1 Participants

To help capture a range of IT professionals, anonymized survey data were collected from an online Qualtrics survey between February and May 2023 from a convenience sample. The cross-sectional study, IT MindState, was advertised on social media accounts (LinkedIn and X/Twitter), recruitment platforms (Prolific and SurveyCircle), and shared via email. To be eligible, respondents needed to have at least one full year of work experience in the IT sector, be at least 18 years old, and be actively working in the US.

#### 2.1.2 Procedures

Electronic voluntary informed consent was obtained from 462 IT MindState respondents. A subsample of 301 respondents completed the burnout assessment questionnaire described in the next section and were the main focus of this study. Prolific participants received the required $8/h, yielding a payment of $1.60 for the 12-min median time for their participation in the survey. All respondents were provided with the option to be randomly selected for one of four $75 gift cards via a separate unlinked survey (for anonymity). The parent study (Protocol #18281) received approval as an exempt study by the home institution's Human Subjects and Institutional Review Board (27). This study employed a comparative, analytical cross-sectional design.

### 2.2 Instrumentation

#### 2.2.1 Outcome measure

Occupational burnout was screened using the Burnout Assessment Tool (BAT), a 12-item questionnaire focused on four core dimensions/symptoms of burnout: exhaustion, mental distance, emotional impairment, and cognitive impairment (28, 29). Response options per question included 1 = “Never” to 5 = “Always.” Exhaustion captures the loss of energy (e.g., *I feel mentally exhausted)*; mental distance is detachment from the work (e.g., *I struggle to find any enthusiasm for my work*); emotional impairment is about not being able to regulate emotions (e.g., *I feel unable to control my emotions*); while cognitive impairment is more about regulating memory and attention (e.g., *I have trouble staying focused*) (28, 29). Final scores are obtained by summing up all scores and dividing by 12, ranging from 1 to 5. Overall burnout scores at 2.54 and above signify a high risk of burnout, and scores above 2.96 refer to severe burnout being experienced. The BAT is a validated measurement tool (alpha mean = 0.85) that is more comprehensive when compared to other burnout measures like the MBI and shows consistency across countries, including the US (26). Each core symptom has its individual clinical at risk cut-off score as follows: Exhaustion is 3.49, mental distancing is 2.16, cognitive impairment is 2.82, and emotional impairment is 2.82. Values at or above the cut-off correspond with being at risk. The BAT-12 scale's reliability was confirmed using Cronbach's alpha (α = 0.92).

##### 2.2.1.1 Secondary outcomes

***Mental Health Literacy in the Workplace (MHL-W)–***The MHL-W comprises four vignettes depicting a hypothetical coworker's behavior in the workplace (30). This is a previously validated instrument (α = 0.95) (30). Each of the four vignettes have four Likert scale questions (1 = very low to 5 = very high) measuring the four distinct MHL concepts: (1) level of knowledge in being able to recognize a specific disorder–“*What might be happening with [him/her]*”, (2) level of knowledge and beliefs regarding risk factors and prevention –“*How you could prevent the situation from becoming worse*”, (3) level of knowledge and attitudes about help-seeking–“*What you should say or do in the situation*”, and (4) level of knowledge and beliefs regarding interventions–“*Resources or services that might be helpful*”. The final MHL-W score was calculated by summing up all 16 questions, with possible scores ranging from 16 to 80. Higher scores correspond with greater self-reported knowledge of mental health. The MHL-W scale's reliability was confirmed using Cronbach's alpha (α = 0.91).

### 2.3 Data analysis

**Figure 1:**
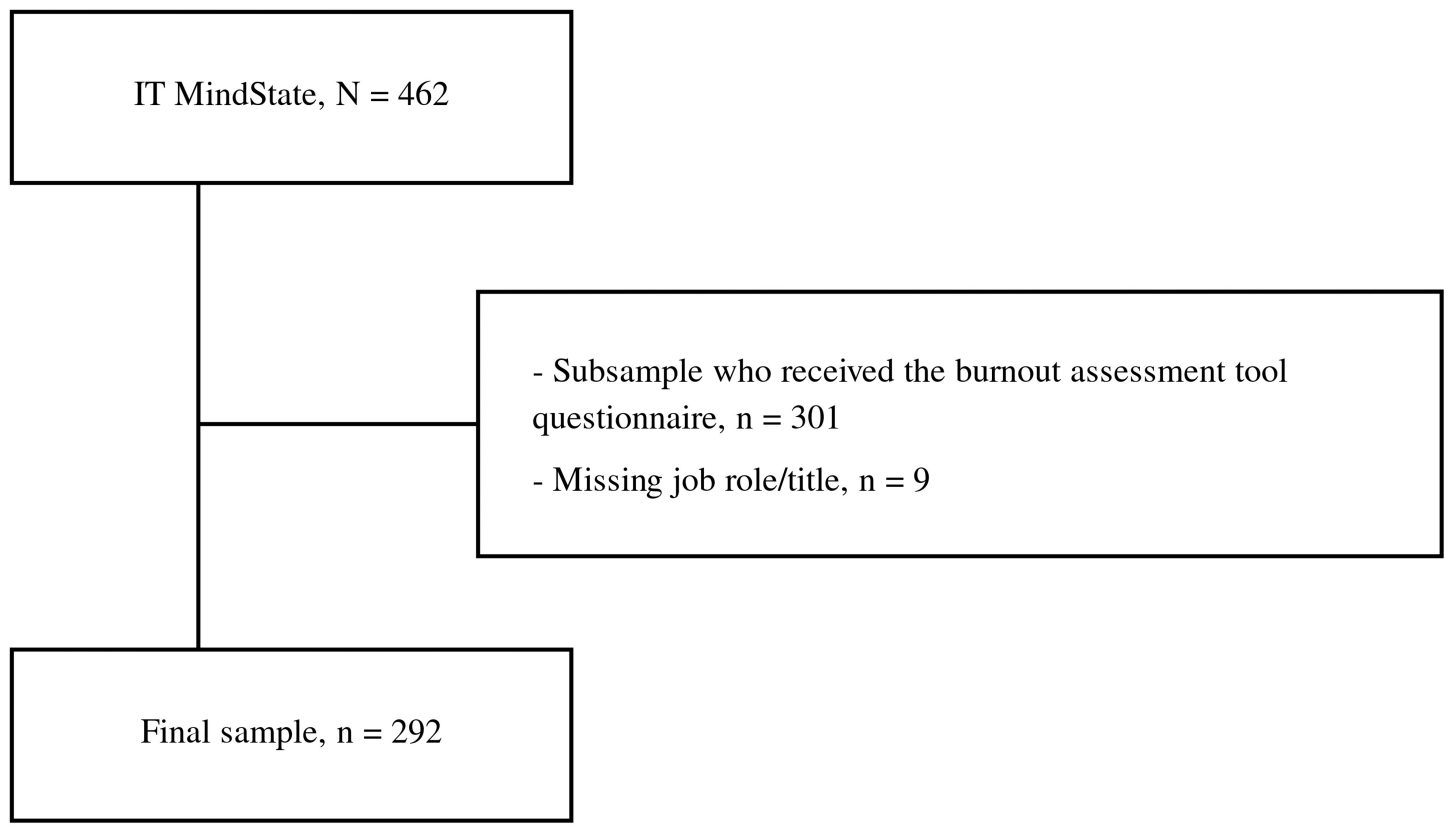
Flowchart of sample inclusion procedure.

This study only considered complete cases for the analysis (*n* = 292). There were nine unreported job roles among the 301 participants who completed the BAT assessment. In Figure 1, we illustrate the sample inclusion procedure.

Descriptive statistics (age, sex, race, job role) and primary study variables were described as means with standard deviations (SD) for continuous variables and frequencies/percentages for categorical data. Graphical techniques, such as histograms were employed to visually assess the distribution of the main variables. The Shapiro-Wilks test and visual inspection revealed the normality assumption was violated, as the scores were significant skewed. Therefore, the Wilcoxon rank-sum test (Mann-Whitney U test) was run to compare burnout, MHL, and help-seeking scores between the two groups because the scores are not normally distributed. Burnout was analyzed in two ways to provide both statistical rigor and clinical relevance. Continuous BAT scores were utilized for hypothesis testing to maximize statistical power and capture the full range of respondent experiences. Additionally scores were dichotomized into at-risk and not-at-risk categories based on established BAT thresholds to facilitate the interpretation of the practical and organizational impact of burnout within the IT workforce (28, 29). Multivariate linear regression was used to examine the relationship between job role and primary outcomes while controlling for sociodemographic covariates (age, sex, race/ethnicity, mental health history, and geographic location). Statistical significance is defined by *p* < 0.05. Stata v19.5 (College Station, TX) was used to analyze the data.

## 3 Results

### 3.1 Descriptive statistics

As shown in Table 1, over two-thirds of the 292 respondents, 77% (*n* = 226), were male and 78% (*n* = 227) were White. All US census geographic regions were represented in the data. Nearly a fifth of the respondents reported their primary geographical job location was in the Northeast, and almost 23% (*n* = 67) were in the South Atlantic. Of the 292, 29% (*n* = 84) shared they were in a management or leadership role (e.g., Executive Director, IT Manager).

**Table 1:** Sociodemographic Characteristics by IT Job Level.

Characteristics	Leadership/Management N = 84 (28.8%)	Associate N = 208 (71.2%)	Total N = 292 (100%)
Age median	40	36	38
Sex			
Female	20 (23.8%)	46 (22.1%)	66 (22.6%)
Male	64 (76.2%)	162 (77.9%)	226 (77.4%)
Race/Ethnicity (*n* = 291)			
African American or Black	5 (6.0%)	10 (4.8%)	15 (5.2%)
American Indian, Alaskan Native, Other Race^*^	6 (7.1%)	9 (4.3%)	15 (5.2%)
Asian or Asian American	9 (10.7%)	25 (12.1%)	34 (11.7%)
White	64 (76.2%)	163 (78.7%)	227 (78.0%)
Prior mental health diagnosis			
Yes	24 (28.6%)	56 (26.9%)	80 (27.4%)
No	60 (71.4%)	152 (73.1%)	212 (72.6%)
Geographic region			
Northeast	21 (25.0%)	37 (17.8%)	58 (19.9%)
Midwest–East North Central	7 (8.3%)	41 (19.7%)	48 (16.4%)
Midwest–West North Central	7 (8.3%)	12 (5.8%)	19 (6.5%)
South Atlantic	18 (21.4%)	49 (23.6%)	67 (22.9%)
East South Central	5 (5.9%)	8 (3.8%)	13 (4.4%)
West South Central	10 (11.9%)	17 (8.2%)	27 (9.2%)
West–Mountain	6 (7.1%)	14 (6.7%)	20 (6.8%)
West–Pacific	10 (11.9%)	30 (14.4%)	40 (13.7%)

^*^Includes selection of more than one race.

### 3.2 Main findings

***Hypothesis 1: IT associates would report higher levels of burnout when compared to IT*** ***leadership/management*.**

As shown in Table 2, the average BAT score for the study sample was 2.12 (SD 0.75). Most respondents (71.2%) were below the cutoff score (< 2.54) for risk of burnout. However, 85 participants had BAT scores equal to or greater than 2.54, signifying a high risk of burnout. Of those 85, nearly half (*n* = 42) were above the 2.96 cutoff score, which signifies they were experiencing severe burnout. However, per the Mann-Whitney U Test, there was no significant difference in overall burnout scores between IT professionals in leadership roles and those in associate roles (*z* = 0.403, *p* =.687). The calculated effect size was negligible (Rank-biserial *r* = 0.024). Burnout levels for IT associates (mean = 2.13, 95% CI 2.03, 2.24) and IT leadership (mean−2.10, 96% CI 1.94, 2.27), were not statistically different.

**Table 2:** Sociodemographic characteristics and mental health literacy and burnout by IT Job Level.

Mental health literacy in the workplace and burnout items	Leadership/ Management Mean (SD)	Associate mean (SD)	*p*–value	95% CI	Cohen's *d*
MHL–W	50.4 (10.5)	44.6 (10.4)	< 0.001	(4.22, 9.22)	0.55
BAT12	2.10 (0.8)	2.13 (0.8)	0.687	(-0.17, 0.23)	0.04
Subscales					
Exhaustion	2.48 (0.91)	2.46 (0.93)	0.846	(-0.26, 0.21)	0.03
Mental distance	2.18 (0.96)	2.31 (0.98)	0.320	(-0.12, 0.37)	0.13
Cognitive impairment	2.17 (0.98)	2.22 (0.87)	0.604	(-0.17, 0.29)	0.07
Emotional impairment	1.58 (0.83)	1.54 (0.82)	0.686	(-0.25, 0.17)	0.05

MHSIS, Mental Help Seeking Intention Scale; MHL-W, Mental Health Literacy at the Workplace; BAT, Burnout Assessment Tool.

After adjusting for the covariates, job role remained a statistically non-significant predictor of burnout (*b* = 0.03, *p* =.768), with an effect size of ß = 0.017 as shown in Table 3.

**Table 3:** Linear regression results for BAT and MHL scores.

Independent variable	BAT *b* (SE)	*P–*value	95% CI	Effect size ß	MHL-W *b* (SE)	*P*–value	95% CI	Effect size ß
Leadership	0.03 (0.09)	0.768	(-0.16, 0.21)	0.017	6.55 (1.28)	< 0.001	(4.04, 9.06)	.288

ß, unstandardized coefficient. Reference group, associates.

Adjusted for age, sex, race/ethnicity, mental health history, and geographic region.

The following describes a deeper dive into the four core symptoms of burnout: exhaustion, mental distance, cognitive impairment, and emotional impairment.

#### 3.2.1 Exhaustion

The average overall exhaustion score was 2.46 (SD 0.93). Most 81% (*n* = 236) IT professionals were not at risk for this core symptom. There was no statistically significant difference between IT associates and leadership/management.

#### 3.2.2 Mental distance

The average overall score for mental distance was 2.27 (SD 0.93). In this category, the respondents nearly split: 49% (*n* = 144) showed no risk and 51% (*n* = 148) were at risk. While IT associates had a slightly higher proportion, 54% (*n* = 112), who were at risk when compared to IT leadership/management 42.9% (*n* = 36), the difference was not statistically significant.

#### 3.2.3 Cognitive impairment

The average overall cognitive impairment score was 2.21 (SD 0.91). With nearly a fourth (*n* = 69) of respondents being at risk in this category, no significant differences were found in this category between IT associates and leadership/management.

#### 3.2.4 Emotional impairment

The average overall score was 1.55 (SD 0.82). Across the sample, the majority, 84% (*n* = 245) were not at risk for this category. With the IT leadership/management group having a higher proportion, 20% (*n* = 17) in the at-risk group when compared to the 14% (*n* = 30) of IT associates, but not statistically significant.

***Hypothesis 2: IT leadership/management would report higher knowledge about mental*** ***health information compared to IT associates*.**

In terms of MHL, the leadership/management group reported a higher average MHL score (50.4) when compared to the associate-level IT professionals, and per the Wilcoxon rank-sum test this is statistically significant (*z* = −4.097, *p* < 0.001). Leadership reported higher mean MHL-W scores (mean = 50.44, 95% CI 48.16, 52.72) compared to their associate counterparts (mean = 44.64, 95% CI 43.20, 46.07). This difference was supported by a medium effect size (*r* =.24).

Job role remained a significant predictor of mental health literacy (*b* = 6.55, *p* < 0.001) after adjusting for covariates, suggesting that the literacy gap between leaders is independent of age or other demographic factors.

### 3.3 Secondary findings

A Wilcoxon (Mann-Whitney) test was performed to examine the relationship between sex and burnout. The relationship between these variables was marginally significant, *P* = 0.054. Male IT professionals were less likely to report increased risk of burnout compared to female IT professionals.

## 4 Discussion

The objectives of this study were to 1) explore burnout among IT professionals and identify any differences between IT professionals in leadership/management roles and those in associate-level roles and 2) compare IT leadership/management and IT associates' assessment of their MHL.

This study found that there was no significant difference in overall burnout scores nor each of the four dimensions of burnout between IT associates and IT leadership/management. However, IT leadership/management scored higher in MHL than IT associates. Therefore, the hypothesis that IT associates would report higher levels of burnout when compared to leadership/management was not supported by the results. Unlike previous studies exploring differences in anxiety, depression and loneliness between IT managers and technicians, there was no significant difference in burnout (13). However, the hypothesis that IT leadership/management would report higher knowledge about mental health information compared to IT associates was supported.

While group differences in burnout demographics did not reach statistical significance, the observed percentage point differences suggest meaningful organizational trends. For example, IT associates 54% (*n* = 112), were at risk in the mental distance category compared to IT leadership/management, 42.9% (*n* = 36). This 11.1 percentage point gap, though not statistically significant in this sample, may reflect a practical disparity in psychological detachment. Associates in IT roles may utilize mental distancing as a primary coping strategy for job demands where they have less decision-making authority. This aversion to work or mental withdrawal from work among IT associates is a critical issue across all job roles and could affect interactions with clients. While for emotional impairment, a larger proportion of IT leadership/management 20% (*n* = 17) were in the at-risk group compared to their associate counterparts 14% (*n* =30). IT leaders are often tasked not only with the technical oversight but with the emotional burden of team collaborations as well as crisis management during system failure, for example. This highlights that while total burnout scores may appear similar, how it actually manifests may differ based on the dimensions of burnout.

The statistically significant difference in MHL between leadership and associates was characterized by a moderate to large effect size (Cohen's *d* = 0.55). The 5.8-point advantage held by leadership suggests a meaningfully higher baseline of knowledge regarding mental health symptoms and resources. However, the practical significance of this gap remains nuanced. While higher MHL among leaders is a necessary precursor for a supportive work environment, our findings indicate that this literacy does not currently translate into lower burnout for leadership themselves. Nor does it necessarily bridge the help-seeking intention gap for associates. MHL was found to be positively associated with leadership roles, though its role as a protective resource against burnout cannot be temporally established by this cross-sectional data. This difference in MHL could be a result of professional development opportunities, training, or leadership experiences that enhance MHL. Further exploration of which trainings are offered and taken by leadership in IT and their experiences may assist in clarifying their reported increased knowledge. Alternatively, this difference in knowledge between the groups might result from who decides to go into leadership in the first place. For instance, it is also possible that individuals with higher MHL are more likely to seek leadership or management roles. Associates in particular are a higher-risk group because although they have similar burnout scores to their leadership counterparts, their knowledge of mental health symptoms and ability to recognize them is lower.

Our multivariable analysis provides critical clarity regarding the factors driving wellbeing in the IT workforce. While the initial bivariate analysis suggested a lack of difference in burnout between leadership and associate, the regression model confirms this null finding is not due to age or experience masking the effect. Instead it appears that burnout in IT is more closely linked to individual factors, rather than to their hierarchical position. Conversely the persistent significance of job role in the MHL model underscores that the literacy gap is a distinct feature of leadership positions in IT, after adjusting for covariates. This suggests that the higher literacy observed in leadership is likely due to role-specific experience, exposure, or training rather than simply being a byproduct of seniority or survivorship in the field.

Our results revealed a significant discrepancy that while IT leadership possessed significantly higher MHL than the IT associates, their burnout scores remained comparable. Theoretically, this suggests that MHL may act as a resource for recognizing distress, but not as a preventative strategy with the ability to reduce job demands. This supports a core component of the JD-R model, that increasing resources (MHL) cannot fully compensate for excessive demands.

Unlike previous studies, which found a higher percentage of burnout reported among women IT professionals compared to their male counterparts (31), this study found the difference to be marginally significant. This difference in findings could be partially explained by the over two-thirds male composition of the study sample, while the prior study had nearly half male and female in its sample.

### 4.1 Implications

Prior literature has assessed burnout with the Maslach Burnout Inventory (MBI) instrument. However, the MBI instrument has its own limitations; therefore, this study aimed to complement previous research with the BAT instrument. BAT addresses the limitations found in the MBI instrument (e.g., cultural validity) and provides a broader view of the burnout experience (28, 29). As previous research has found that assessing burnout as exhaustion alone is insufficient (23), the BAT instrument generates both an overall burnout score and across its four dimensions. This approach can help identify which burnout areas individuals are most at risk for and, therefore, help create or adopt workplace interventions specifically for IT professionals to reduce burnout.

### 4.2 Limitations

There are some limitations to this study. First, this is a convenience sample; therefore, the results are not generalizable to the entire IT profession. We acknowledge that the recruitment strategy was efficient for reaching a broad geographic range of IT professions, though this may have introduced selection bias. Individuals with burnout experiences could be either over- or under-represented in this sample. For instance, those with extreme burnout experiences might have had a higher motivation to participate or could have been too exhausted or no longer working within IT to engage with the study (non-response bias). Future research should employ stratified random sampling to capture those missed experiences. Second, given the cross-sectional nature of this study: we cannot determine the directionality of the relationships observed. For instance, while we observed higher MHL in leadership, it remains unclear whether leadership training enhances literacy or if individuals with higher MHL are more likely to be promoted into leadership roles. This study also does not capture the physical factors (e.g., sedentary) that are descriptive of the work being conducted, which could also help uncover other factors contributing to the pressures at work.

Although the sample (77.4% male vs. 22.6% female) resembled the demographic characteristics of the IT professional workforce overall by sex (24% women) (32), this sample is overrepresented by white respondents (78%) when compared to industry benchmarks. The 2024 CompTIA industry report estimates the technology workforce is comprised of approximately 58% white IT professionals (33). While the primary goal of this study was to conduct an exploratory assessment of the relationships between job role, MHL, and burnout outcomes, the homogeneity of the current sample is a notable limitation. Specifically, the findings should be interpreted as primarily reflecting the experiences of white male professionals in this sample. Future research should utilize targeted sampling to capture the unique burnout trajectories of women as well as racial and ethnic minorities in the IT sector.

Furthermore, this study utilized broad categorizations for IT roles (leadership vs. associate), which may have masked differences in IT sub-specialties. It is important to interpret the non-significant differences in burnout between leadership and associates with caution. A *post-hoc* power analysis revealed that with the current sample size and variance, the study was underpowered to detect small to moderate effect sizes. Therefore, the observed mean difference in BAT scores may represent a lack of statistical power rather than a true absence of occupational differences. This is further observed by the marginal significant difference in burnout by sex. Similarly, while geographic data was collected, the prevalence of remote work in IT sector suggests that their location may not fully capture the organizational culture or economic stressors of their employment, especially when working remotely. Future research should employ granular role-based sampling and account for work-arrangements to understand these complex dynamics.

The literacy gap observed suggests an opportunity for intervention studies. Specifically, research should evaluate whether implementing MHL training for associates reduces burnout or enhances help-seeking behaviors. Finally, future work should examine how specific IT job demands relate to the distinct burnout dimensions of cognitive and emotional impairment. Such research is important for moving from general awareness to evidence-based, role-specific organizational interventions.

While exploratory, this study underscores that burnout in IT is a complex, industry-wide challenge that is not restricted to any single hierarchical level. By identifying the specific MHL gap between leadership and associates, this work provides a foundation for the multivariable, longitudinal, role-specific research required to protect the wellbeing of the modern IT workforce.

## Data Availability

The raw data supporting the conclusions of this article will be made available by the authors, without undue reservation.
